# Diagnostic value of integrated ^18^F-FDG PET/MRI for staging of endometrial carcinoma: comparison with PET/CT

**DOI:** 10.1186/s12885-022-10037-0

**Published:** 2022-09-01

**Authors:** Yang Yu, Le Zhang, Bilkis Sultana, Bo Wang, Hongzan Sun

**Affiliations:** 1 Department of Radiology, Shengjing Hospital of China Medical University, Sanhao Street No36, Heping District, Shenyang, 110004 China; 2 Department of Nuclear Medicine, Shengjing Hospital of China Medical University, Shenyang, 110004 China; 3 Liaoning Provincial Key Laboratory of Medical Imaging, Shenyang, 110004 China; 4 Department of Radiology, Qingdao Municipal Hospital, Qingdao University, Qingdao, 266011 China

**Keywords:** Endometrial carcinoma, PET/MRI, PET/CT, FIGO stage

## Abstract

**Purpose:**

To explore the diagnostic value of integrated positron emission tomography/magnetic resonance imaging (PET/MRI) for the staging of endometrial carcinoma and to investigate the associations between quantitative parameters derived from PET/MRI and clinicopathological characteristics of endometrial carcinoma.

**Methods:**

Altogether, 57 patients with endometrial carcinoma who underwent PET/MRI and PET/computed tomography (PET/CT) preoperatively were included. Diagnostic performance of PET/MRI and PET/CT for staging was compared by three readers. Associations between PET/MRI quantitative parameters of primary tumor lesions and clinicopathological characteristics of endometrial carcinoma were analyzed. Histopathological results were used as the standard.

**Results:**

The overall accuracy of the International Federation of Gynecology and Obstetrics (FIGO) staging for PET/MRI and PET/CT was 86.0% and 77.2%, respectively. PET/MRI had higher accuracy in diagnosing myometrial invasion and cervical invasion and an equivalent accuracy in diagnosing pelvic lymph node metastasis against PET/CT, although without significance. All PET/MRI quantitative parameters were significantly different between stage I and stage III tumors. Only SUV_max_/ADC_min_ were significantly different between stage I and II tumors. No parameters were significantly different between stage II and III tumors. The SUV_max_/ADC_min_ in the receiving operating characteristic (ROC) curve had a higher area under the ROC curve for differentiating stage I tumors and other stages of endometrial carcinoma.

**Conclusions:**

PET/MRI had a higher accuracy for the staging of endometrial carcinoma, mainly for FIGO stage I tumors compared to PET/CT. PET/MRI quantitative parameters, especially SUV_max_/ADC_min_, were associated with tumor stage and other clinicopathological characteristics. Hence, PET/MRI may be a valuable imaging diagnostic tool for preoperative staging of endometrial carcinoma.

## Introduction

In 2020, 417,367 new endometrial carcinoma cases and 97,370 endometrial carcinoma-related deaths were reported worldwide [1]. The treatment modalities for endometrial carcinoma include surgery, radiotherapy, chemotherapy, and hormone therapy, and the choice of treatment depends on the staging. In 1971, the International Federation of Gynecology and Obstetrics (FIGO) Committee sought to improve the clinical staging of endometrial carcinoma. These staging criteria were last revised in 2009 [2]. Preoperative imaging techniques have exceptional advantages for staging endometrial carcinoma because of their non-invasiveness and relative accuracy. Compared with clinical staging, these imaging techniques can accurately evaluate staging before treatment, which plays a significant role in guiding clinical treatment and judging prognosis.

Magnetic resonance imaging (MRI) and positron emission tomography/computed tomography (PET/CT) are two standard imaging techniques that are clinically applied for the preoperative staging of endometrial carcinoma. Compared with MRI, PET/CT plays a key role in exploring lymph node metastasis. PET/CT has been reported to have high sensitivity, specificity, and positive predictive value for endometrial carcinoma metastasis [3]. However, PET/CT has a relatively low resolution for soft tissues and thus, lower efficiency in detecting the primary tumor of the endometrial carcinoma compared with MRI [4]. Recently, PET/MRI has attracted attention as a new multi-modality molecular imaging technique. PET/MRI integrates the high precision of MRI and high sensitivity of PET by generating complete integrated images at the structural, functional, and molecular levels [5]. PET/MRI is reported to have value in evaluating gynecological tumor staging, assessing treatment response, detecting recurrent lesions, and predicting prognosis [6–8]. Moreover, previous studies have demonstrated the relationship between MR quantitative parameters and endometrial carcinoma staging and pathological features [9, 10]. However, the value of PET/MRI parameters in endometrial carcinoma staging has not yet been analyzed.

Thus, this study aimed to evaluate the applicability of integrated PET/MRI in diagnosing and staging endometrial carcinoma by comparing PET/MRI and PET/CT imaging results with postoperative pathological data. Furthermore, we extracted quantitative parameters from PET/MRI to explore their relationship with the staging and clinicopathological characteristics of endometrial carcinoma.

## Material and methods

### Patients

Data of patients with pathologically proven endometrial carcinoma, who underwent PET/CT and PET/MRI from December 2017 to January 2021 were retrospectively analyzed. All procedures were approved by the ethics committee of the hospital. Written informed consent was obtained from all patients.

**Fig. 1:**
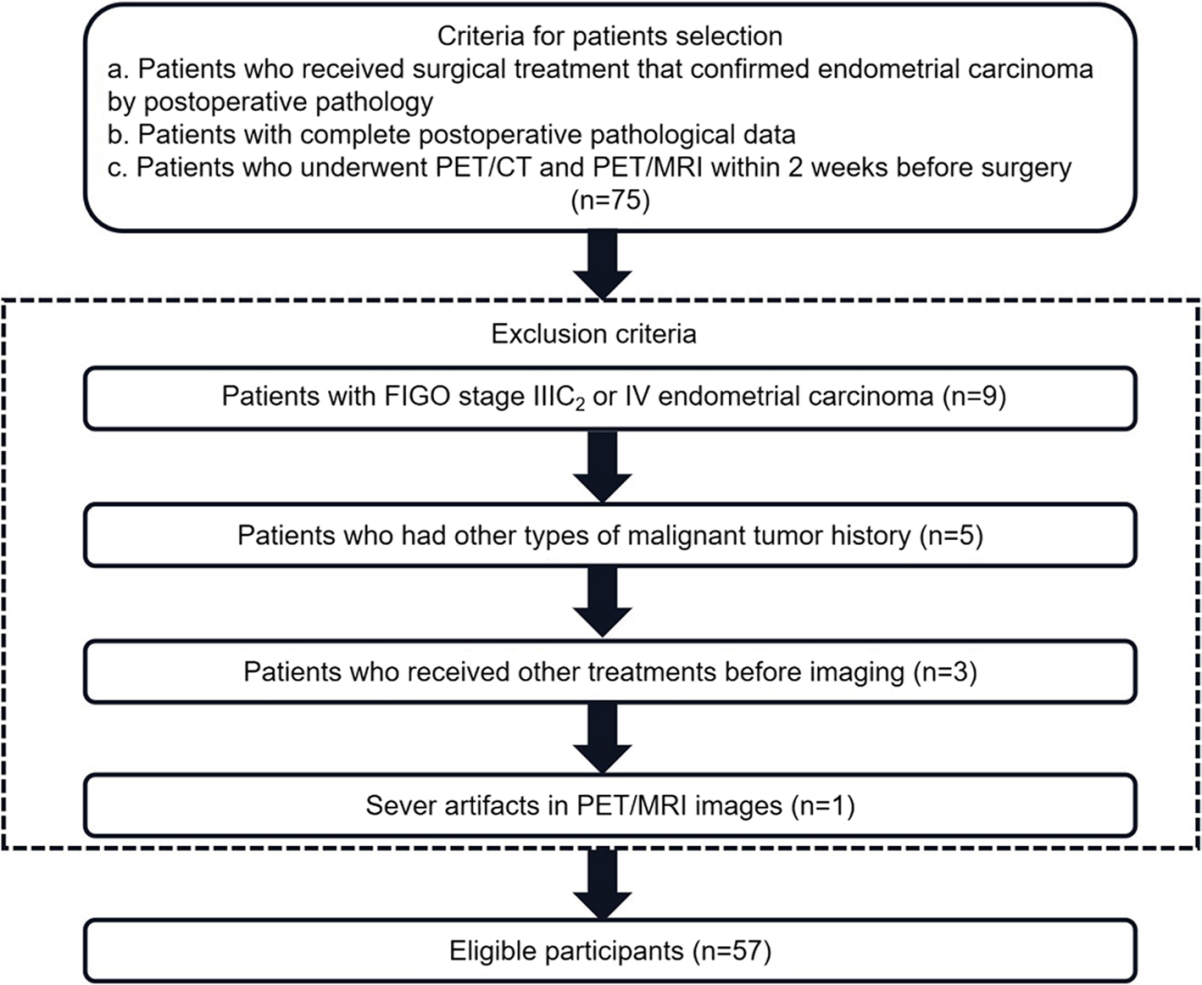
Flowchart of patient inclusion and exclusion criteria

We screened the patients based on the following inclusion criteria: (1) patients who underwent PET/CT and PET/MRI before treatment, (2) patients who received surgical treatment that confirmed endometrial carcinoma by postoperative pathology, (3) patients with complete postoperative pathological data, and (4) patients who had < 2 weeks between imaging and surgery. The exclusion criteria were as follows: (1) patients who received other treatments (e.g., radiotherapy and chemotherapy) before imaging, (2) patients who had other types of malignant tumor history before endometrial carcinoma diagnosis, (3) patients in whom the measurement of parameters was affected by artifacts in PET/MRI images, and (4) patients who belonged to the FIGO stage IIIC_2_ or IV. Finally, 57 patients were enrolled in this study (Fig. 1). All 57 patients underwent total abdominal or laparoscopic hysterectomy with bilateral salpingo-oophorectomy, and 41 patients underwent pelvic lymphadenectomy.

### PET/CT scanning and image acquisition

Before non-contrast-enhanced ^18^F-fluorodeoxyglucose (^18^F-FDG) PET/CT scanning, all patients fasted for ≥ 6 h and had blood glucose levels of ≤ 7 mmol/L. They were injected with 3.70–5.55 MBq/kg ^18^F-FDG in the resting state. After 60 min, ^18^F-FDG PET/CT scanning was performed with a Discovery PET/CT 690 scanner (GE Healthcare, Waukesha, WI, USA) while the patients were lying on the scan bed. The scan ranged from the calvarium to the middle thigh (120 s/bed). The slice thickness, tube voltage, and tube current for CT scans were 3.75 mm, 120–140 keV, and 80 mA, respectively.

### PET/MRI scanning and image acquisition

Pelvic ^18^F-FDG PET/MRI scanning was performed 33 ± 12 min after PET/CT scanning. All images were acquired from the scans that were performed using Signa PET/MRI (GE Healthcare, Waukesha, WI, USA), integrating time-of-flight–PET, and 3.0 T MRI (GE Signa 750w) scanners. PET images and MRI images were acquired simultaneously. A 32-channel coil (upper anterior array) served as the cavitary scanning coil. The pelvic axial scan ranged from the vaginal level to the superior iliac boundary. For pelvic MRI scanning, T2-weighted images (T2WI) were acquired in the axial, sagittal, and coronal planes using the following T2WI parameters: repetition time (TR), 2600–3400 ms; echo time (TE), 60–90 ms; section thickness, 5 mm; interval, 1 mm; matrix, 384 × 384; field of view (FOV), 240 × 240 mm. Axial T1-weighted images parameters were as follows: TR, 500 ms; TE, 8 ms; section thickness, 5 mm; interval, 1 mm; matrix, 384 × 384; FOV, 240 × 240 mm. Axial diffusion-weighted imaging (DWI) with b-values of 0 and 800 s/mm^2^ were obtained. For PET scanning, the correction for γ-ray attenuation was performed with the Dixon MRI sequence. PET scanning was conducted under list mode. Images were reconstructed by iterative ordered subset expectation maximization. The pelvic scan time for PET/MRI was approximately 24 min.

### Image analysis

All ^18^F-FDG PET/CT and PET/MRI images were uploaded to a GE AW4.6 workstation (GE Healthcare, Waukesha, WI, USA) and then post-processed using PET volume computer-assisted reading (VCAR) software. Three radiologists/nuclear medicine physicians, each with double board certifications and with > 5 years of medical imaging diagnosis experience, evaluated the PET/CT and PET/MRI images independently and sequentially at two time points. PET/MRI image evaluation was performed 4–5 weeks after PET/CT image evaluation, thereby eliminating subjective image bias. The readers were unaware of the other readers’ evaluation results. To achieve a final summary of the results, diagnosis was determined through negotiation and consensus when their opinions were inconsistent.

For maximum standardized uptake value (SUV_max_) measurements of PET/MRI, by applying the iterative adaptive algorithm, PET VCAR enables automatic segmentation. SUV_max_ was defined as the maximum value of SUV.

IMAgenGINE MRToolbox (Vision Tech, Hefei, Anhui, China) software was used for apparent diffusion coefficient (ADC) measurements. Regions of interest (ROIs) were drawn on the ADC maps with T1WI and T2WI sequences as references. ROIs were manually delineated on the slices containing the largest diameter of the tumors as much as possible, avoiding necrotic and hemorrhage tissue. Necrotic areas were defined as areas with relatively low signal intensity on DWI and T1WI, and high signal intensity on T2WI compared with solid tumors. Hemorrhage areas were defined as areas with relatively high signal intensity on DWI and T1WI compared with solid tumors. ADC_mean_ and ADC_min_ were defined as the average and the lowest ADC values in ROI, respectively. Three readers independently measured all parameters, and the average was calculated.

### Diagnostic criteria

PET/CT and PET/MRI diagnostic criteria for staging endometrial carcinoma were based on the FIGO staging system. Postoperative pathological staging was applied as the gold standard.

To diagnose myometrial invasion, the proportion of the tumor thickness that invaded the myometrial layer was used to calculate the myometrial invasion. The superficial myometrial invasion ratio was < 50%. The deep myometrial invasion ratio was ≥ 50%. The diagnosis of cervical invasion was based on high uptake tumor invasion at the cervix in the PET/CT image, low signal disappearance of the cervical interstitial layer under the T2WI sequence, and high signal in the DWI image at the cervix. On PET/MRI images, tumor invasion of adjacent structures was determined primarily on the basis of MRI findings, with reference to PET findings. On PET/CT images, tumor invasion of adjacent structures was determined primarily on the basis of PET findings, with reference to CT findings.

On PET/CT and PET/MRI images, lymph node metastasis diagnosis was based on the abnormally high uptake of FDG in the pelvic lymph nodes (i.e., exceeding normal muscle or exceeding normal lymph nodes at the same level in the contralateral pelvis that corresponded to the lymph node chains), regardless of whether their short-axis diameter was higher than 1 cm. In addition, abnormally high signal intensity on DWI was also considered as a positive sign of lymph node metastasis.

### Histological examination

Tumor staging was performed according to the 2018 FIGO criteria. All surgical specimens were examined and reported by a gynecologic pathologist. A comprehensive histological evaluation was performed for each lesion, including histological type, tumor grade, myometrial and cervical stromal invasion, and lymph node status. For patients who did not undergo pelvic lymph node dissection, lymph node metastasis was confirmed at follow-up (at least 12 months). A decrease in lymph node size after treatment was considered a sign of malignancy.

### Statistical analysis

The McNemar test was used to determine the statistical significance of differences in the accuracy of staging as determined by PET/MRI and PET/CT. The inter-reader agreement in PET/CT and PET/MR evaluation from the three readers was assessed using Kendall’s concordance coefficient (W). The associations between PET/MRI parameters and clinicopathological characteristics were analyzed using independent sample t-test (two sets of variables) and one-way analysis of variance, followed by Bonferroni posthoc test (three sets of variables). The correlations between PET/MRI parameters were assessed using Pearson’s correlation coefficient test. Receiver operating characteristic (ROC) curve was used to evaluate the value of PET/MRI quantitative parameters to differentiate FIGO stage I and FIGO stage II + III endometrioid carcinoma. The Youden index was used to obtain the cut-off value. The SPSS 22.0 software (IBM Corp., Armonk, NY, USA) was used for all statistical analyses. Statistical significance was set at *p* < 0.05.

## Results

### Patient characteristics

Altogether, 57 patients were enrolled in the study. The participants’ clinicopathological characteristics are summarized in Table 1.

**Table 1:** Clinicopathological characteristics of patients

**Characteristics**	**Value**
**No. of patients**	57
**Age (years)**	
**Range**	38–75
**Mean ± SD**	57 ± 8
**Histological type**	
**Endometrioid adenocarcinoma**	49 (86.0%)
**Non-endometrioid adenocarcinoma**	8 (14.0%)
**Tumor grade**	
**G1 + G2**	40 (70.2%)
**G3**	17 (29.8%)
**FIGO stage**	
**IA**	30 (52.6%)
**IB**	7 (12.3%)
**II**	9 (15.8%)
**IIIA**	2 (3.5%)
**IIIB**	1 (1.8%)
**IIIC**_1_	8 (14.0%)
**Myometrial invasion depth**	
**< 50%**	39 (68.4%)
**≥ 50%**	18 (31.6%)
**Cervical invasion**	
**Absent**	41 (71.9%)
**Present**	16 (28.1%)
**Metastatic pelvic lymph node**	
**Negative**	49 (86.0%)
**Positive**	8 (14.0%)

### Comparison between PET/MRI and PET/CT staging

The diagnostic results of PET/MRI and PET/CT staging for endometrial carcinoma are presented in Tables 2 and 3. The overall accuracy of FIGO staging for PET/MRI and PET/CT was 86.0% (49/57) and 77.2% (44/57), respectively (no significant difference, *P* = 0.180). PET/MRI overstaged the actual FIGO stage in five (8.8%) patients, whereas PET/CT overstaged the actual FIGO stage in nine (15.8%) patients. PET/MRI incorrectly classified two IA tumors as IB, one IA tumor as II, and one IA tumor and one II tumor as IIIC_1_. PET/CT incorrectly classified six IA tumors as IB, one IA tumor as II, and one IA tumor and one II tumor as IIIC_1_. PET/MRI understaged the actual FIGO stage in three (5.3%) patients, whereas PET/CT understaged the actual FIGO stage in four (7.0%) patients. PET/MRI incorrectly classified one IB tumor as IA, one II tumor as IB, and one IIIC_1_ tumor as II. PET/CT incorrectly classified two IB tumors as IA, one II tumor as IB, and one IIIC_1_ tumor as II. Kendall’s concordance coefficient showed that evaluation of PET/CT and PET/MR images was highly concordant among the three readers (W = 0.883, *p* < 0.001; W = 0.915, *p* < 0.001, respectively).

**Table 2:** Comparison between PET/MRI staging and pathological staging

**PET/MRI staging**	**Pathological staging**					
	IA	IB	II	IIIA	IIIB	IIIC_1_
**IA**	26	1	0	0	0	0
**IB**	2	6	1	0	0	0
**II**	1	0	7	0	0	1
**IIIA**	0	0	0	2	0	0
**IIIB**	0	0	0	0	1	0
**IIIC**_**1**_	1	0	1	0	0	7
**Total**	30	7	9	2	1	8
**Accuracy**	86.7% (26/30)	85.7% (6/7)	77.8% (7/9)	100%(2/2)	100% (1/1)	87.5% (7/8)

**Table 3:** Comparison between PET/CT staging and pathological staging

**PET/CT staging**	**Pathological staging**					
	IA	IB	II	IIIA	IIIB	IIIC_1_
**IA**	22	2	0	0	0	0
**IB**	6	5	1	0	0	0
**II**	1	0	7	0	0	1
**IIIA**	0	0	0	2	0	0
**IIIB**	0	0	0	0	1	0
**IIIC**_**1**_	1	0	1	0	0	7
**Total**	30	7	9	2	1	8
**Accuracy**	73.3% (22/30)	71.4% (5/7)	77.8% (7/9)	100% (2/2)	100% (1/1)	87.5% (7/8)

**Fig. 2:**
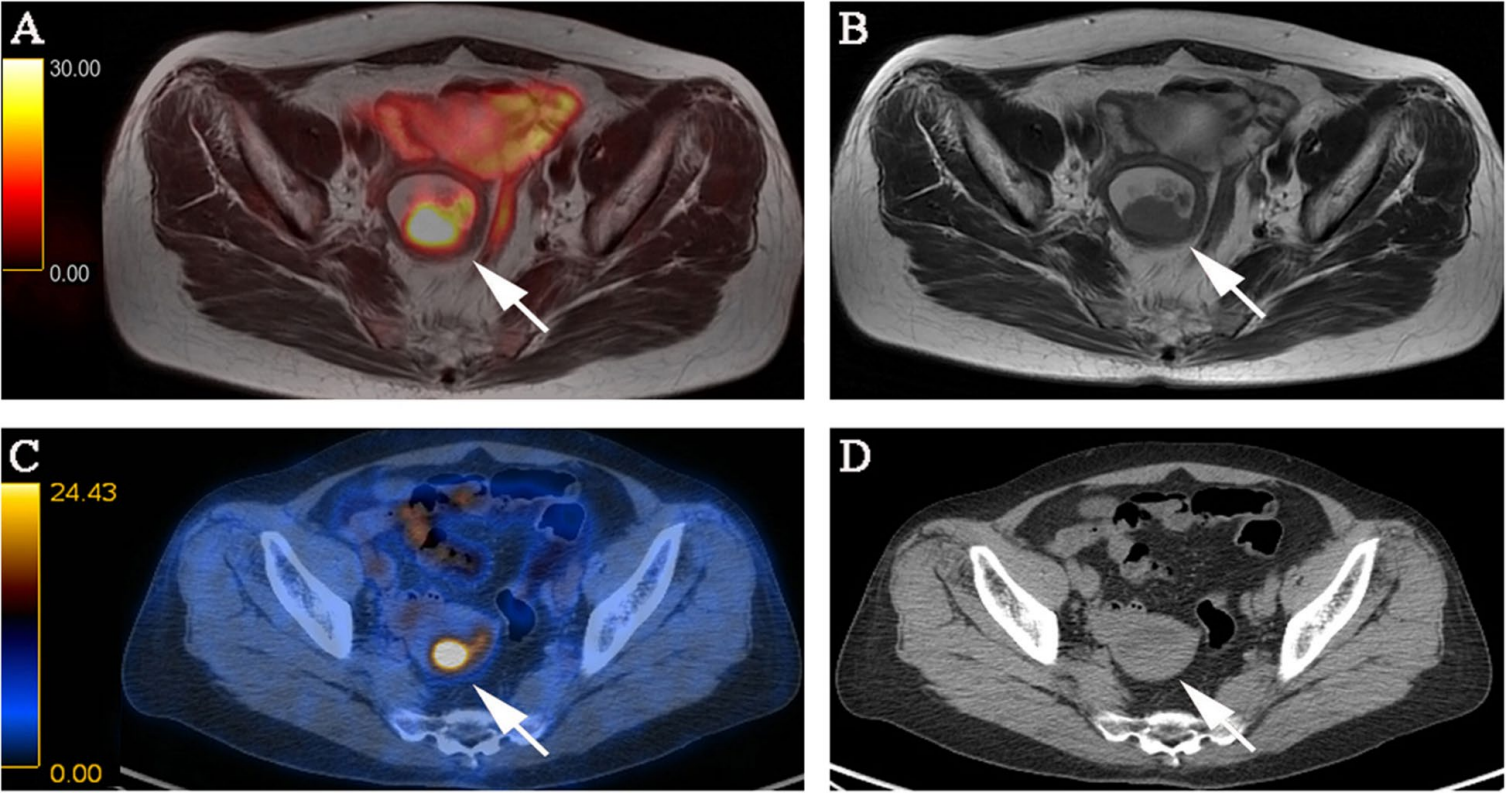
FIGO stage IB, pathologically confirmed as highly differentiated adenocarcinoma in a 64-year-old female patient. **A** PET/MR images. **B** MR T2-weighted images. **C** PET/CT images. **D** CT images. PET/MRI and MRI show the endometrial carcinoma invading > 50% of the myometrium. PET/CT shows the endometrial carcinoma invading < 50% of the myometrium, while CT is difficult to show the border of the tumor. The PET/MRI staging was consistent with the pathological stage, namely FIGO stage IB. The PET/CT staging was IA. The white arrow indicates endometrial carcinoma. FIGO, International Federation of Gynecology and Obstetrics; PET, positron emission tomography; MRI, magnetic resonance imaging; CT, computed tomography

**Fig. 3:**
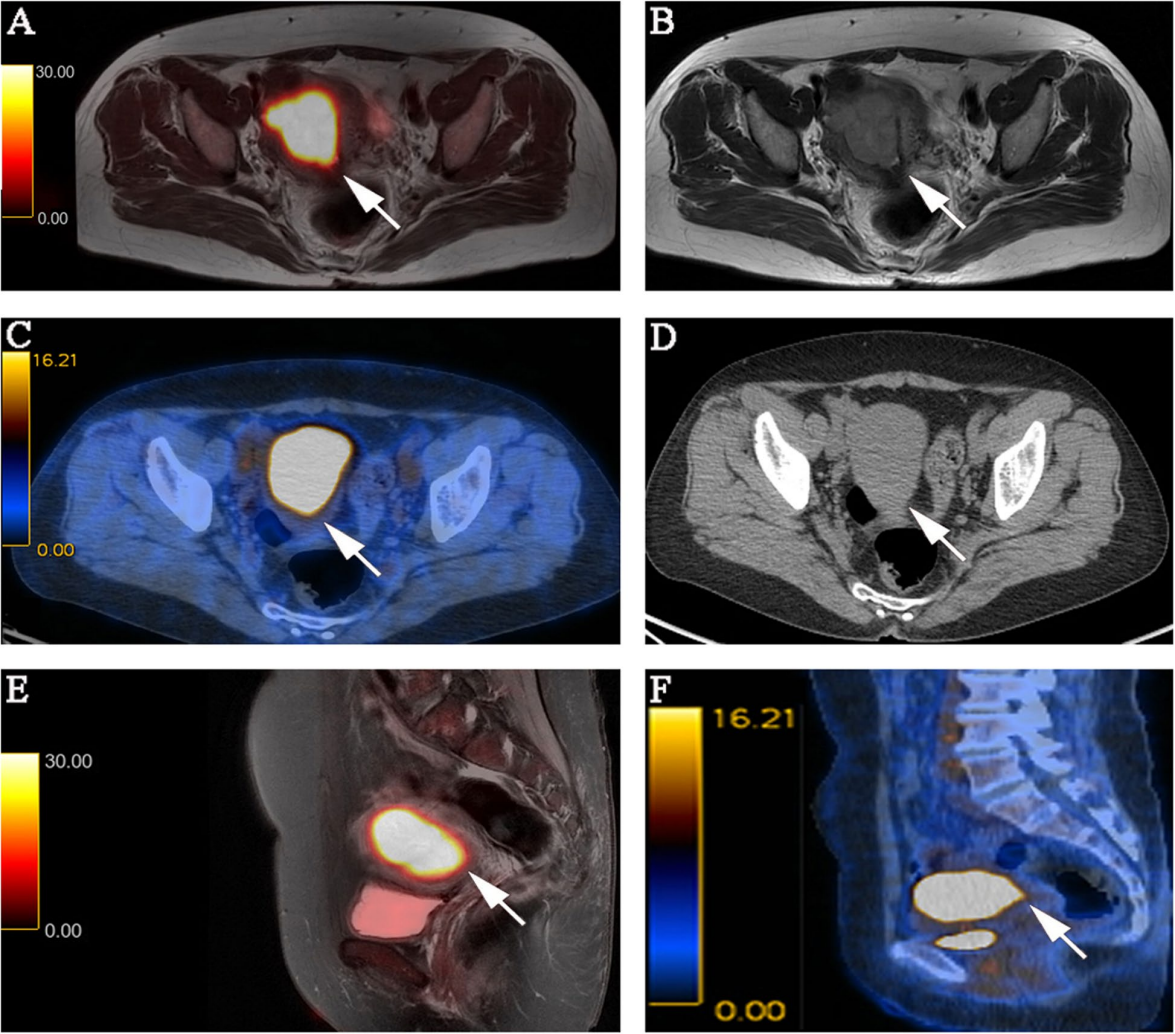
FIGO stage II pathologically confirmed moderately differentiated adenocarcinoma in a 57-year-old female patient. **A** Axial PET/MR images. **B** Axial MR T2-weighted images. **C** Axial PET/CT images. **D** Axial CT images. **E** Sagittal PET/MR images. **F** Sagittal PET/CT images. PET/MRI and MRI show the endometrial carcinoma invading the cervical stroma. PET/CT also shows the endometrial carcinoma invading the cervical stroma, while CT is difficult to determine the extension of the tumor. The PET/MRI and PET/CT staging were consistent with the pathological stage, namely FIGO stage II. The white arrow indicates endometrial carcinoma. FIGO, International Federation of Gynecology and Obstetrics; PET, positron emission tomography; MRI, magnetic resonance imaging; CT, computed tomography

**Fig. 4:**
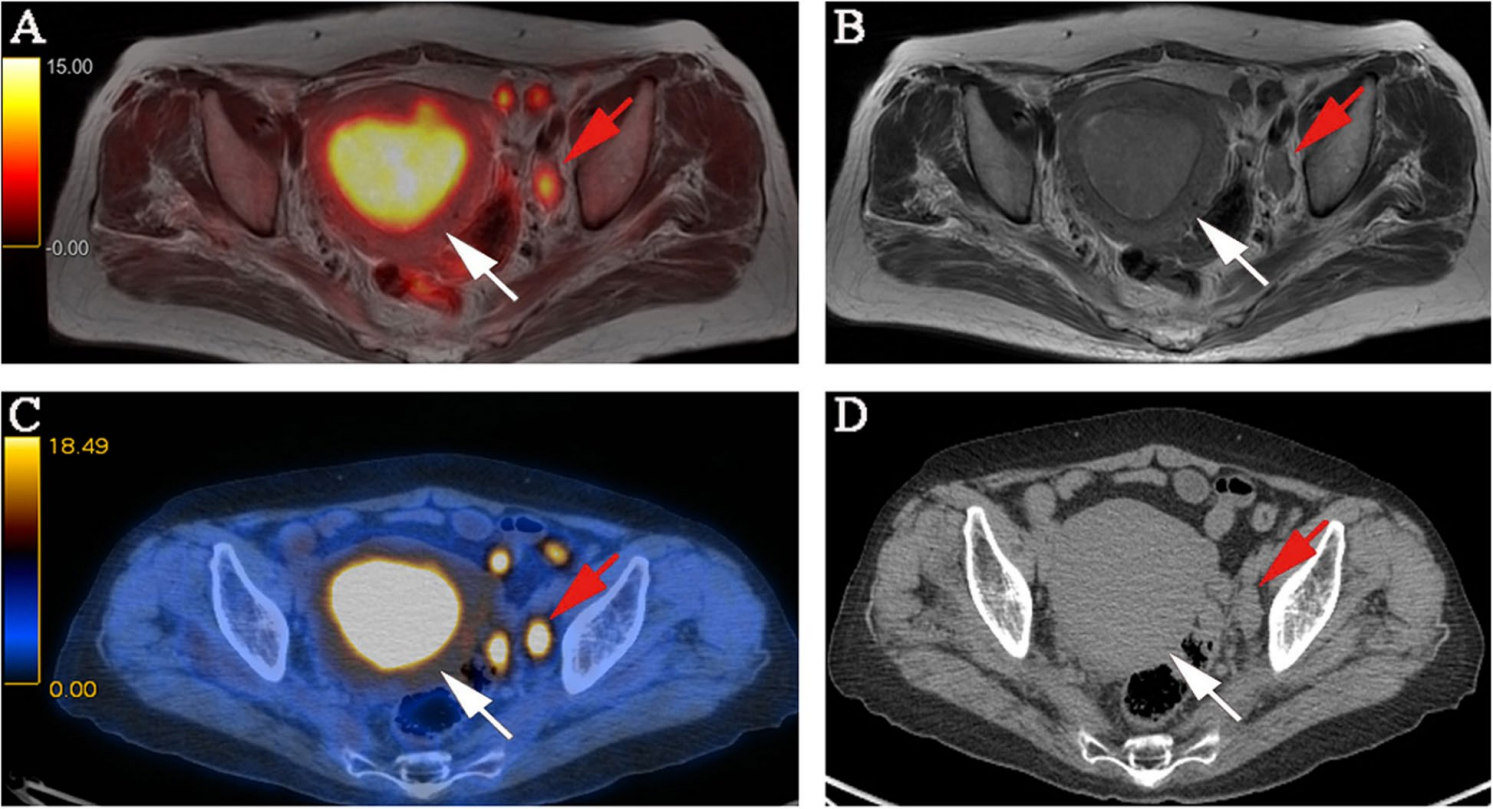
FIGO stage IIIC_1_, pathologically confirmed moderately differentiated adenocarcinoma in a 48-year-old female patient. **A** PET/MR images. **B** MR T2-weighted images. **C** PET/CT images. **D** CT images. Both PET/MRI and PET/CT show swelling of the left pelvic lymph node and high FDG uptake. The PET/MRI and PET/CT staging were consistent with the pathological stage, namely FIGO stage IIIC_1_. The white arrow indicates endometrial carcinoma, and the red arrow indicates metastatic lymph node. FIGO, International Federation of Gynecology and Obstetrics; PET, positron emission tomography; MRI, magnetic resonance imaging; CT, computed tomography

The sensitivity, specificity, and accuracy for detecting myometrial invasion were 88.9%, 94.9%, and 93.0% for PET/MRI and 61.1%, 79.5%, and 73.7% for PET/CT, respectively (Table 4; no significant difference, *P* = 1). PET/MRI incorrectly estimated the depth of myometrial invasion in four tumors, whereas PET/CT incorrectly estimated the depth of myometrial invasion in 10 tumors. Figure 2 depicts the detection of myometrial invasion. The sensitivity, specificity, and accuracy for detecting cervical invasion were 81.3%, 95.1%, and 91.2% for PET/MRI, and 81.3%, 92.7%, and 89.5% for PET/CT, respectively (Table 4; no significant difference, *P* = 1). PET/MRI incorrectly estimated cervical invasion in five tumors, whereas PET/CT incorrectly estimated cervical invasion in six tumors. For one case of stage IIIC_1_ tumor, PET/CT incorrectly classified the absence of cervical invasion as the presence of cervical invasion. Meanwhile, PET/MRI classification was correct, although this did not affect the FIGO stage of the tumor. Figure 3 depicts the detection of cervical invasion. In the patient-based analysis, the sensitivity, specificity, and accuracy for detecting pelvic lymph node metastasis were 87.5%, 95.9%, and 94.7% for both PET/MRI and PET/CT, respectively (Table 4; no significant difference, *P* = 1). Both PET/MRI and PET/CT incorrectly diagnosed pelvic lymph node metastasis in three tumors. For one case of stage IA tumor and one case of stage II tumor, both PET/MRI and PET/CT had false-positive results, with abnormally high uptake values in pelvic lymph nodes, although the pathological results were negative. For one case of stage IIIC_1_ tumor, both PET/MRI and PET/CT had a false-negative result. Figure 4 depicts the detection of pelvic lymph node metastasis.

**Table 4:** Comparison of PET/MRI and PET/CT for patient-based myometrial invasion depth, cervical invasion, and metastatic pelvic lymph node

	**PET/MRI**	**PET/CT**
**≥ 50% myometrial invasion**		
**Sensitivity**	88.9% (16/18)	61.1% (11/18)
**Specificity**	94.9% (37/39)	79.5% (31/39)
**Accuracy**	93.0% (53/57)	73.7% (42/57)
**Cervical invasion**		
**Sensitivity**	81.3% (13/16)	81.3% (13/16)
**Specificity**	95.1% (39/41)	92.7% (38/41)
**Accuracy**	91.2% (52/57)	89.5% (51/57)
**Metastatic pelvic lymph node**		
**Sensitivity**	87.5% (7/8)	87.5% (7/8)
**Specificity**	95.9% (47/49)	95.9% (47/49)
**Accuracy**	94.7% (54/57)	94.7% (54/57)

### Associations between PET/MRI quantitative parameters and clinicopathological characteristics

According to Table 5, SUV_max_, ADC_mean_, ADC_min_, SUV_max_/ADC_mean_, and SUV_max_/ADC_min_ showed a significant difference between stage I and stage III tumors (*P* < 0.001–0.021). Only SUV_max_/ADC_min_ exhibited a significant difference between stage I and stage II tumors (*P* = 0.043). No parameters demonstrated a significant difference between stage II and stage III tumors.

**Table 5:** Associations between PET/MRI quantitative parameters and clinicopathological characteristics

	**SUV**_**max**_	**ADC**_**mean**_ **(× 10**^**−6**^**mm**^**2**^**/s)**	**ADC**_**min**_ **(× 10**^**−6**^**mm**^**2**^**/s)**	**SUV**_**max**_**/ADC**_**mean**_	**SUV**_**max**_**/ADC**_**min**_
**FIGO stage**					
**I**	15.41 ± 6.88	0.90 ± 0.14	0.61 ± 0.13	17.36 ± 8.12	26.00 ± 12.07
**II**	18.95 ± 4.67	0.84 ± 0.05	0.51 ± 0.10	22.52 ± 5.62	37.81 ± 9.79
*** p*-value**	0.500	0.551	0.059	0.311	0.043*
**I**	15.41 ± 6.88	0.90 ± 0.14	0.61 ± 0.13	17.36 ± 8.12	26.00 ± 12.07
**III**	21.99 ± 7.77	0.79 ± 0.08	0.46 ± 0.06	28.17 ± 10.78	48.37 ± 15.75
*** p*-value**	0.020*	0.021*	0.001*	0.001*	< 0.001*
**II**	18.95 ± 4.67	0.84 ± 0.05	0.51 ± 0.10	22.52 ± 5.62	37.81 ± 9.79
**III**	21.99 ± 7.77	0.79 ± 0.08	0.46 ± 0.06	28.17 ± 10.78	48.37 ± 15.75
*** p*-value**	0.968	0.928	0.931	0.416	0.200
**Histological type**					
**E**	17.58 ± 7.52	0.88 ± 0.13	0.58 ± 0.12	20.56 ± 9.73	32.05 ± 15.66
**NE**	15.20 ± 4.24	0.83 ± 0.10	0.49 ± 0.16	18.46 ± 6.01	32.98 ± 13.67
*** p*-value**	0.389	0.335	0.084	0.558	0.875
**Tumor grade**					
**G1 + G2**	16.16 ± 7.13	0.89 ± 0.14	0.59 ± 0.14	18.71 ± 9.31	29.43 ± 15.51
**G3**	19.78 ± 6.81	0.83 ± 0.08	0.52 ± 0.09	23.92 ± 8.38	38.65 ± 12.96
*** p*-value**	0.082	0.048*	0.075	0.052	0.036*
**Myometrial invasion depth**					
**< 50%**	16.28 ± 7.66	0.89 ± 0.14	0.59 ± 0.14	18.99 ± 10.25	29.46 ± 15.80
**≥ 50%**	19.32 ± 5.63	0.84 ± 0.07	0.52 ± 0.08	23.03 ± 6.11	38.07 ± 12.57
*** p*-value**	0.139	0.077	0.021*	0.128	0.047*
**Cervical invasion**					
**Absent**	16.47 ± 7.84	0.89 ± 0.14	0.60 ± 0.13	19.14 ± 10.17	28.96 ± 15.37
**Present**	19.23 ± 4.73	0.84 ± 0.06	0.49 ± 0.09	23.13 ± 5.85	40.44 ± 11.88
*** p*-value**	0.194	0.060	0.003*	0.147	0.010*
**Metastatic pelvic lymph node**					
**Negative**	16.37 ± 6.59	0.88 ± 0.13	0.58 ± 0.13	18.95 ± 8.07	29.67 ± 13.70
**Positive**	22.56 ± 8.73	0.81 ± 0.08	0.48 ± 0.07	28.31 ± 12.56	47.53 ± 16.38
*** p*-value**	0.022*	0.145	0.030*	0.007*	0.002*

* indicates *p* < 0.05

Tumors with a myometrial invasion depth ≥ 50% had higher SUV_max_/ADC_min_ and lower ADC_min_ than those with a myometrial invasion depth < 50% (*P* = 0.047 and 0.021, respectively). Tumors with cervical invasion had higher SUV_max_/ADC_min_ and lower ADC_min_ than those without cervical invasion (*P* = 0.010 and 0.003, respectively). Tumors with positive pelvic lymph node metastasis had higher SUV_max_, SUV_max_/ADC_mean_, and SUV_max_/ADC_min_ than those with negative pelvic lymph node metastasis (*P* = 0.022, 0.007, 0.002, respectively). Tumors with positive pelvic lymph node metastasis had lower ADC_min_ than those with negative pelvic lymph node metastasis (*P* = 0.030). ADC_mean_ and SUV_max_/ADC_min_ indicated a significant correlation with tumor grade (*P* = 0.048 and 0.036, respectively). No significant correlations were identified between PET/MRI quantitative parameters and histological type. No correlation was identified between SUV_max_ and ADC_mean_ (*P* = 0.390) as well as between SUV_max_ and ADC_min_ (*P* = 0.479).

### PET/MRI quantitative parameters for staging of endometrial carcinoma

**Fig. 5:**
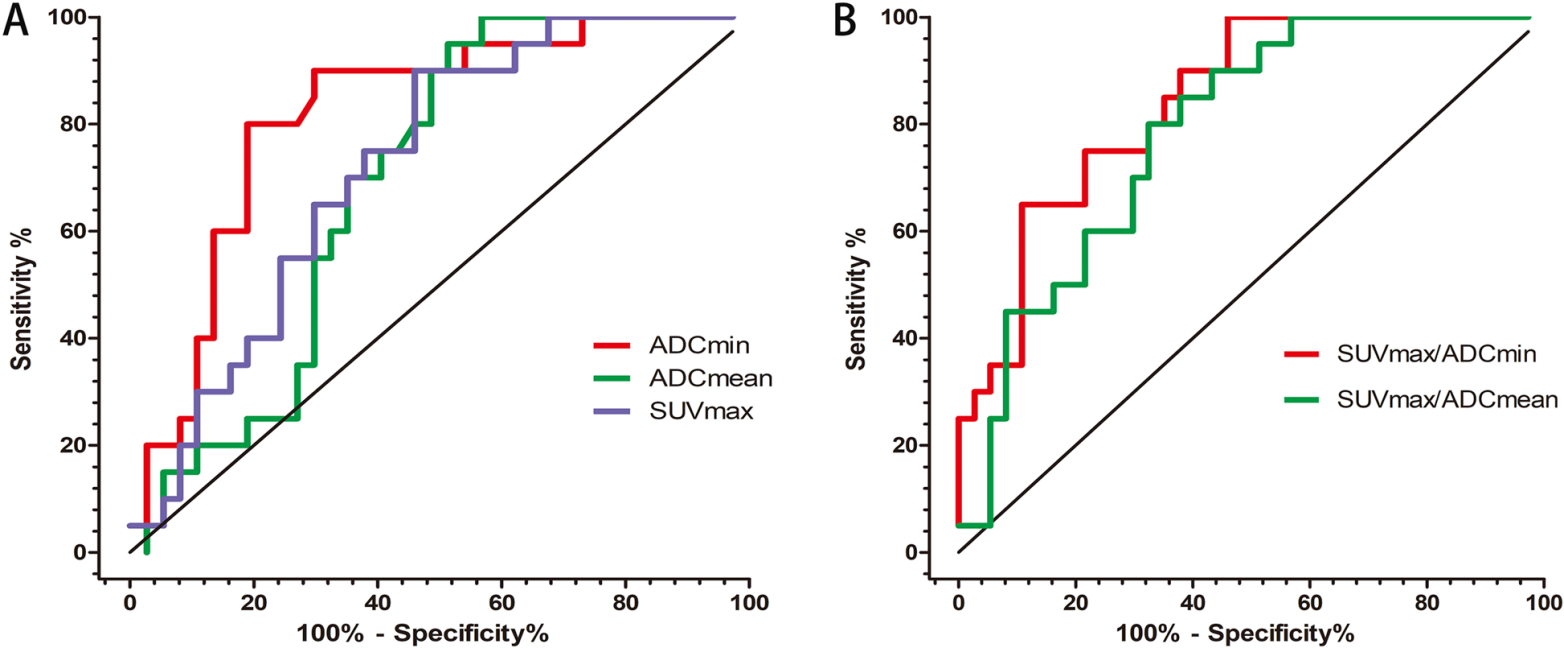
Receiver operating characteristic curve analysis for PET/MRI quantitative parameters to differentiate FIGO stage I and FIGO stage II + III endometrial carcinoma. FIGO, International Federation of Gynecology and Obstetrics; PET, positron emission tomography; MRI, magnetic resonance imaging

ROC curve results of SUV_max_, ADC_mean_, ADC_min_, SUV_max_/ADC_mean_, and SUV_max_/ADC_min_ for differentiating FIGO stage I and FIGO stage II + III endometrial carcinoma are listed in Table 6. SUV_max_/ADC_min_ had a higher area under the curve (AUC) for differentiating the FIGO stage of endometrial carcinoma than other PET/MRI parameters (AUC = 0.843, 95% CI = 0.744–0.943, *P* < 0.001). The cut-off value of SUV_max_/ADC_min_ was 37.16 according to the Youden index. Tumor SUV_max_/ADC_min_ of ≥ 37.16 had a sensitivity of 65% and specificity of 89% for differentiating the endometrial carcinoma’s FIGO stage. The ROC curves of SUV_max_, ADC_mean_, ADC_min_, SUV_max_/ADC_mean_, and SUV_max_/ADC_min_ are depicted in Fig. 5.

**Table 6:** Diagnostic performance of PET/MRI quantitative parameters for differentiating FIGO stage I and FIGO stage II + III endometrial carcinoma

	**SUV**_**max**_	**ADC**_**mean**_	**ADC**_**min**_	**SUV**_**max**_**/ADC**_**mean**_	**SUV**_**max**_**/ADC**_**min**_
**AUC (95% CI)**	0.724(0.594–0.854)	0.695(0.561–0.829)	0.817(0.703–0.931)	0.786(0.670–0.903)	0.843(0.744–0.943)
***p*-value**	0.006*	0.016*	< 0.001*	< 0.001*	< 0.001*

* indicates *p* < 0.05

## Discussion

In comparing the values of PET/MRI and PET/CT for staging in patients with endometrial carcinoma, PET/MRI had higher accuracy for FIGO stage I tumors, while the accuracy was similar in stage II and III tumors. Regarding the associations between PET/MRI quantitative parameters and tumor stage and other clinicopathological characteristics, SUV_max_/ADC_min_ was correlated with more clinicopathological characteristics. Therefore, PET/MRI can be used as an effective diagnostic tool for preoperative staging of endometrial carcinoma.

Pathological examination is the gold standard for endometrial carcinoma staging. However, such pathological examinations can be traumatic to patients. Moreover, making an accurate diagnosis of tumor invasion depth and adjacent organ invasion can be difficult. Therefore, precise staging based on imaging has received greater research attention in recent years. Integrated ^18^F-FDG PET/MRI can provide the high-density resolution of soft tissues and useful functional information (e.g., metabolism and diffusion). Thus, PET/MRI has been applied in studies of clinical gynecological malignancies [11–14].

Our results revealed that PET/MRI staging, which was performed using an integrated PET/MRI scanner, had an excellent diagnostic performance for endometrial carcinoma. PET/MRI had higher diagnostic accuracy for stage I tumors than PET/CT. The diagnosis of stage I endometrial carcinoma is mainly associated with the depth of myometrial invasion. Compared with CT and PET, MRI-T2WI can display the cervix, the junctional zone, the myometrial layer, and endometrial zonal structures in all layers, which is essential for evaluating early-stage endometrial cancer. The technical principle that underlies DWI involves detecting and comparing the diffusion velocity of water molecules between cancer cells and normal cells, which can identify signs of T2WI sequence interference that is caused by irregular endometrial hyperplasia. DWI can complement routine sequences to further improve the advantages of MRI positioning [15, 16]. Building on the merits of T2WI and DWI, integrated PET/MRI is advantageous over PET/CT for diagnosing the myometrial invasion of endometrial carcinoma. Bian et al. have reported that integrated PET/MRI proved significantly more accurate for detecting myometrial invasion than PET/CT (81.8% vs. 45.9%). This is similar to our results, although the former study did not allow patients to simultaneously undergo PET/MRI and PET/CT [17]. Moreover, Tsuyoshi et al. have reported that the accuracy of PET/MRI for detecting myometrial invasion was slightly higher than that of contrast-enhanced MRI (88.9% vs. 86.1%) [18].

The PET/MRI staging accuracy for stages II and III tumors has been observed to be similar to that of PET/CT. Stages II and III are distinguished mainly according to tumor invasion location and lymph node metastasis. Our study indicated that the accuracy of PET/MRI for detecting cervical invasion was slightly higher than that of PET/CT, whereas both PET/MRI and PET/CT were equally accurate in identifying pelvic lymph node metastasis. PET can provide metabolic information and, therefore, has significant advantages in detecting metastatic lesions. Similar to our results, Kitajima et al. have revealed that the accuracy of fused PET/MRI in diagnosing lymph node metastasis was equivalent to that of PET/CT (96.7%) [4]. Moreover, Stecco et al. have reported that on a per-patient basis, fused PET/MRI had the same accuracy (85.1%) as PET/CT in detecting lymph node metastasis. Meanwhile, on a per-node basis, the accuracy of PET/MRI was greater than that of PET/CT (91.2% vs. 87%) owing to the high sensitivity of DWI [19]. Hence, DWI may increase the sensitivity of PET for diagnosing lymph node metastasis.

In addition to morphological staging, PET/MRI can also provide various functional parameters for tumor assessment. Previous studies have demonstrated the correlations between ADC values and clinicopathological characteristics of endometrial carcinoma such as tumor stage, tumor grade, tumor invasion location, and lymph node metastasis [9, 10, 20–22]. Recently, Gai et al. have reported that SUV_max_ derived from PET/CT was correlated with FIGO staging, tissue grading, depth of myometrial invasion, and lymph node metastasis [23]. However, these studies only used SUV or ADC values alone, our study used integrated PET/MRI to explore the associations between functional parameters and clinicopathological characteristics. Here, SUV_max_/ADC_min_ correlated with clinicopathological characteristics of endometrial carcinoma more strongly than any other PET/MRI parameter. Furthermore, the ROC curve demonstrated that the SUV_max_/ADC_min_ resulted in higher AUC for differentiating stage I tumors and other stages of endometrial carcinoma. It should be emphasized that the SUV values of the previous study [23] were obtained from PET/CT images, while the SUV values of our study were obtained from PET/MRI images. For the same patient, SUVmax measured from PET/CT and PET/MRI images are likely to have some differences, possibly due to the effects of the attenuation correction method and the PET scanner. This may lead to differences between our results and those from previous reports [24, 25]. PET and DWI can reflect the biological information of tumors from two aspects of metabolism and diffusion, respectively. Higher SUV values and lower ADC values generally reflect the more aggressive biological behavior of malignant tumors [26, 27]. Therefore, multiparametric PET/MR imaging may help to better understand tumor biology and help to personalize treatment plans and monitor treatment effects.

This study had some limitations. First, since we only performed pelvic ^18^F-FDG PET/MRI scanning, patients with stage IIIC_2_ and stage IV carcinoma were not included. Therefore, the diagnostic value of PET/MRI for distant metastasis could not be evaluated. Second, a certain interval between the PET/CT and PET/MRI examinations was present, which may have affected the results, including the SUV value and PET signal. Third, due to the limitations of the software, we could not perform ADC measurements on the entire tumor lesions, but only selected the slices containing the largest tumor diameter for measurements. This could have lead to inaccurate measurement results. Finally, the number of our patients was limited, and larger sample sizes will be required for future studies.

## Conclusions

PET/MRI had higher accuracy for endometrial carcinoma staging, mainly for FIGO stage I tumors. This was mainly owing to PET/MRI having higher diagnostic accuracy for detecting the depth of myometrial invasion. Moreover, among all the measured PET/MRI quantitative parameters, SUV_max_/ADC_min_ had the strongest association with clinicopathological characteristics. Thus, PET/MRI may be a more valuable diagnostic tool to replace traditional imaging modalities for preoperative staging in patients with endometrial carcinoma. Our findings may help physicians in selecting more appropriate individualized treatment plans for patients.

## Data Availability

The datasets used and/or analyzed during the current study available from the corresponding author on reasonable request.

## Abbreviations

FIGO: International Federation of Gynecology and Obstetrics
MRI: Magnetic resonance imaging
PET/CT: Positron emission tomography/computed tomography
18F-FDG: 18F-fluorodeoxyglucose
T2WI: T2-weighted images
TR: Repetition time
TE: Echo time
FOV: Field of view
DWI: Diffusion-weighted imaging
VCAR: Volume computer-assisted reading
SUVmax: Maximum standardized uptake value
ADC: Apparent diffusion coefficient; ROI: region of interest
AUC: Area under the curve
